# The peanut root exudate increases the transport and metabolism of nutrients and enhances the plant growth-promoting effects of *burkholderia pyrrocinia* strain P10

**DOI:** 10.1186/s12866-023-02818-9

**Published:** 2023-03-30

**Authors:** Lizhen Han, Hong Zhang, Xue Bai, Biao Jiang

**Affiliations:** College of Life Sciences, Guizhou University, 550025 Guiyang, Guizhou China

**Keywords:** *Burkholderia pyrrocinia*, *Arachis hypogaea*, PGPR, Root exudates, Growth-promoting mechanisms

## Abstract

**Background:**

*Burkholderia pyrrocinia* strain P10 is a plant growth-promoting rhizobacterium (PGPR) that can substantially increase peanut growth. However, the mechanisms and pathways involved in the interaction between *B. pyrrocinia* P10 and peanut remain unclear. To clarify complex plant–PGPR interactions and the growth-promoting effects of PGPR strains, the *B. pyrrocinia* P10 transcriptome changes in response to the peanut root exudate (RE) were elucidated and the effects of RE components on biofilm formation and indole-3-acetic acid (IAA) secretion were analyzed.

**Results:**

During the early interaction phase, the peanut RE enhanced the transport and metabolism of nutrients, including carbohydrates, amino acids, nitrogen, and sulfur. Although the expression of flagellar assembly-related genes was down-regulated, the expression levels of other genes involved in biofilm formation, quorum sensing, and Type II, III, and VI secretion systems were up-regulated, thereby enabling strain P10 to outcompete other microbes to colonize the peanut rhizosphere. The peanut RE also improved the plant growth-promoting effects of strain P10 by activating the expression of genes associated with siderophore biosynthesis, IAA production, and phosphorus solubilization. Additionally, organic acids and amino acids were identified as the dominant components in the peanut RE. Furthermore, strain P10 biofilm formation was induced by malic acid, oxalic acid, and citric acid, whereas IAA secretion was promoted by the alanine, glycine, and proline in the peanut RE.

**Conclusion:**

The peanut RE positively affects *B. pyrrocinia* P10 growth, while also enhancing colonization and growth-promoting effects during the early interaction period. These findings may help to elucidate the mechanisms underlying complex plant–PGPR interactions, with potential implications for improving the applicability of PGPR strains.

**Supplementary Information:**

The online version contains supplementary material available at 10.1186/s12866-023-02818-9.

## Background

Peanut (*Arachis hypogaea* L.) is a nutrient-rich legume that is also the sixth most important source of oil and the third most important source of vegetable protein worldwide [1]. The application of large quantities of chemical fertilizers significantly increases peanut production, but it also results in serious environmental pollutions. Thus, the harmful effects of chemical fertilizers on the environment may be avoided by using plant-growth promoting rhizobacteria (PGPR) [2]. Earlier research revealed that PGPR promote plant growth in the following ways: (I) by dissolving phosphorus and potassium in the soil and fixing nitrogen, thereby promoting the uptake and use of nutrients by plants; (II) by synthesizing plant hormones [e.g., indole-3-acetic acid (IAA), cytokinin, gibberellin, and ethylene) that regulate plant growth; and (III) by increasing plant resistance to stresses and protecting against harmful microorganisms [3]. However, plants also affect PGPR strains in the rhizosphere. Previous research demonstrated that plant–microbe interactions involving root exudates (REs) and the chemotactic response of soil microbes to the root-secreted organic compounds play an important role in root colonization [4, 5]. Some RE components may function as signaling molecules that regulate the rhizosphere microbial activity [6–8]. In addition, up to 40% of photosynthetically fixed carbon is released by plant roots in the form of exudates and secretions, lysates, and mucilages and then serve as a carbon and energy source for rhizosphere microorganisms [9]. Accordingly, REs also influence the colonization and growth-promoting effects of PGPR added to the soil. The analysis of the *Pseudomonas aeruginosa* PA01 transcriptome profile revealed that sugar beet REs affect the expression of genes related to metabolism, chemotaxis, and other processes in this bacterial strain [10]. Similar studies were reported in *Bacillus amyloliquefaciens* and *B. subtilis* regulated by the REs of banana and rice seedlings, respectively [11, 12]. The research on the regulatory effects of plant REs on bacterial gene expression has primarily involved *Bacillus* and *Pseudomonas* spp. Moreover, most of these studies focused on stable plant–microbe interactions and relatively few studies have examined the response of other PGPR species to REs. The mechanisms involved in plant host–PGPR interactions in general and the groundnut–PGPR interaction in particular remain to be comprehensively characterized [13]. To further enhance the beneficial effects of PGPR on crops, plant–PGPR interactions should be more thoroughly clarified by applying omic and system biology approaches [14].

The genus *Burkholderia* comprises species that can proliferate in a broad range of ecological niches and are well-known plant-associated bacteria. Several *Burkholderia* species have been identified as PGPR for diverse plants, including tomato, amaranth, maize, rice, and sugarcane [15]. We previously revealed that the plant growth-promoting effects of *Burkholderia pyrrocinia* strain P10 are associated with its ability to solubilize phosphorus, secrete siderophores, and produce indole 3-acetic acid (IAA) as well as 1-aminocyclopropane-1-carboxylate (ACC) deaminase [16]. Strain P10 can effectively colonize the roots and stems of peanut and significantly enhance peanut seedling growth under normal and saline conditions [17, 18]. Unfortunately, the effects of peanut roots on this *B. pyrrocinia* strain are unknown, especially during the early interaction phase. Therefore, the *B. pyrrocinia* P10 transcriptome and growth-promoting effects were analyzed following a peanut RE treatment to clarify the molecular mechanism underlying the interaction between strain P10 and the peanut RE. The results of this study may elucidate clearly the growth-promoting mechanisms of P10 strain, and lay the foundation for exploiting this strain to improve peanut cultivation.

## Results

### Effects of different peanut RE concentrations on the growth of strain P10

**Fig. 1:**
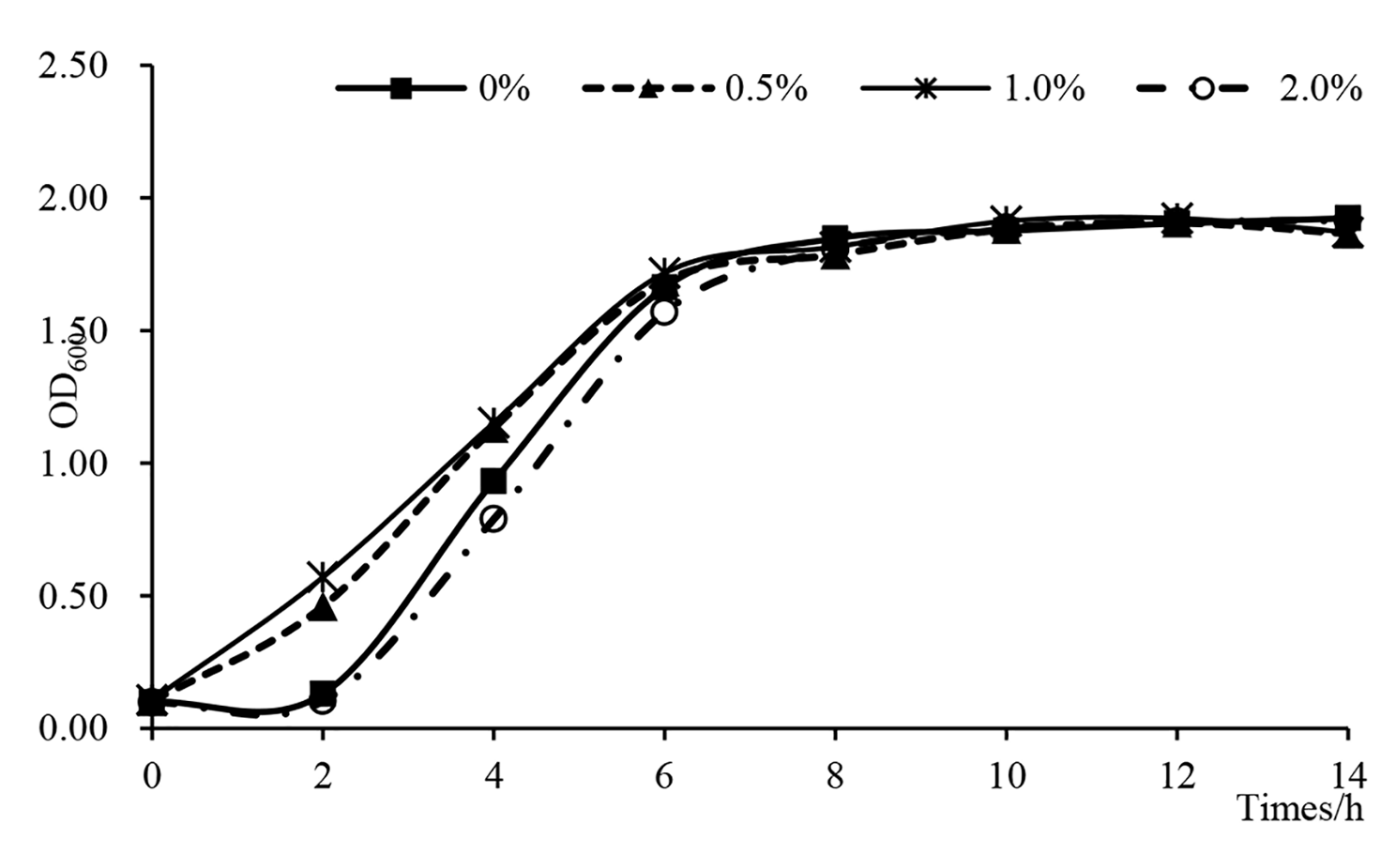
Effects of different peanut RE concentrations on the growth of *Burkholderia pyrrocinia* P10. The OD_600_ value of the strain P10 culture was determined at 2 h intervals during a 14-h incubation

Different peanut RE concentrations differentially affected the growth of *B. pyrrocinia* P10 (Fig. 1). Compared with the effects of the higher concentrations, the lower concentrations significantly promoted the growth of strain P10, which entered the logarithmic growth phase relatively quickly. After a 2-h incubation, the optical density at 600 nm (OD_600_) was significantly higher for the culture supplemented with 0.5–1.0% peanut RE than for the culture lacking peanut RE. This incubation period corresponded to the early interaction phase as well as the early stage of the logarithmic growth phase of strain P10. Therefore, we selected the P10 culture supplemented with 1.0% RE (P10_RE) incubated for 2 h as the treatment group and the P10 culture without RE (P10_N) at the same time-point as the control group for the transcriptome sequencing analysis, which was performed to examine the effects of the peanut RE on the growth and other characteristics of strain P10.

### Analysis of the differentially expressed genes (DEGs) in strain P10 treated with the peanut RE

**Fig. 2:**
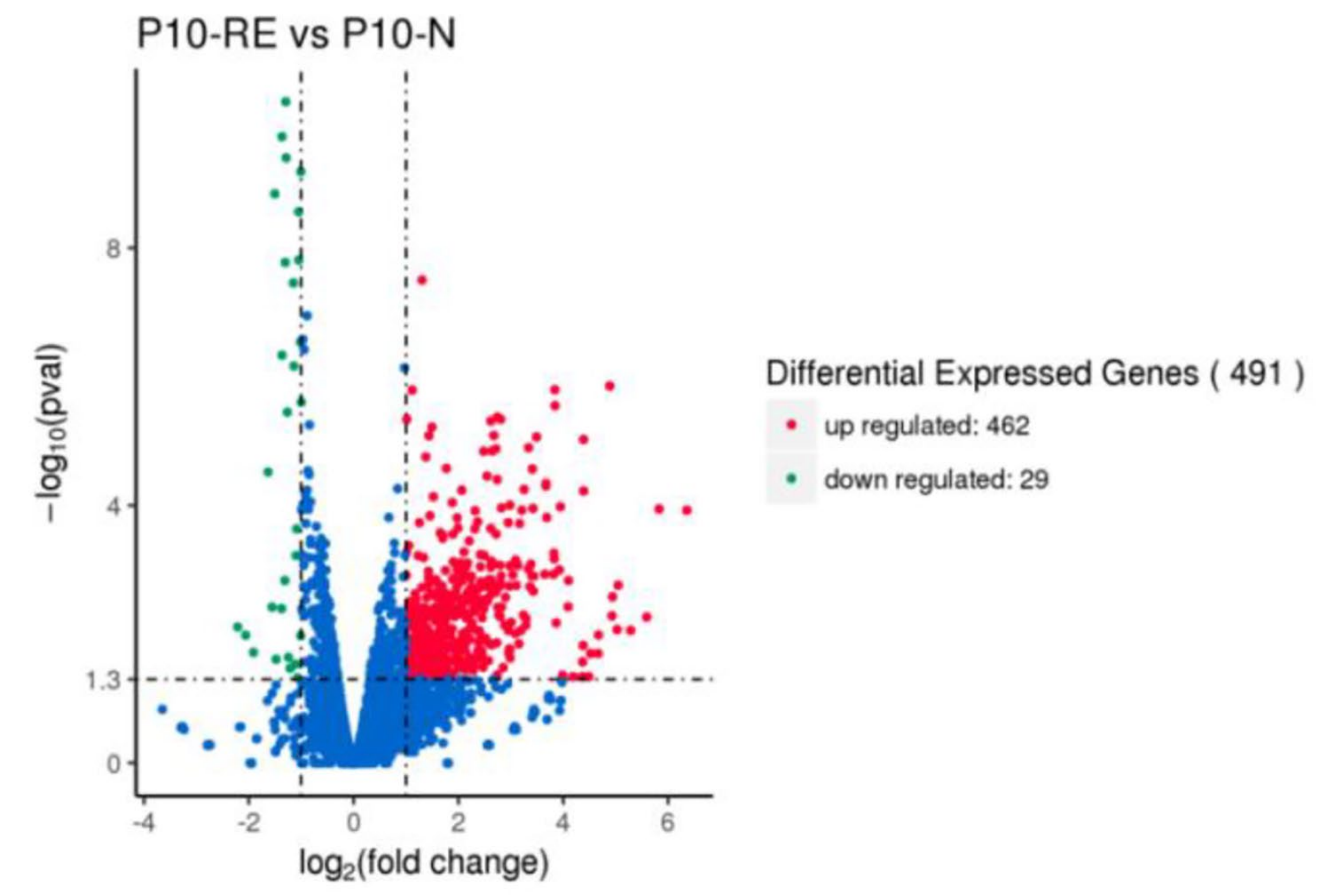
Volcano plot of the DEGs in peanut RE-treated *Burkholderia pyrrocinia* P10. The DEGs were analyzed using DESeq2 (version 1.18.0) in the Bioconductor software package. Red and blue dots represent up-regulated and down-regulated genes, respectively. The abscissa presents the fold-change in gene expression among samples, whereas the ordinate presents the significant differences in gene expression. P10_RE represents the treatment group (i.e., 1% root exudate in the culture medium), whereas P10_N represents the control group (i.e., no root exudate in the culture medium)

For the transcriptome sequencing analysis, approximately 1.78 Gb clean reads were obtained after the quality control step. The Q30% of the libraries ranged from 93.97 to 94.43%, while the clean reads percentage ranged from 88.02 to 89.44% (Supplementary Table S1). Accordingly, the RNA-seq data quality was appropriate for the subsequent analysis. The RNA-seq dataset indicated the expression of 491 genes in strain P10 was affected by the peanut RE, of which 462 genes (94.09%) were up-regulated and 29 genes (5.91%) were down-regulated (Fig. 2).

### Analysis of the enriched kyoto encyclopedia of genes and genomes (KEGG) pathways among the DEGs

**Fig. 3:**
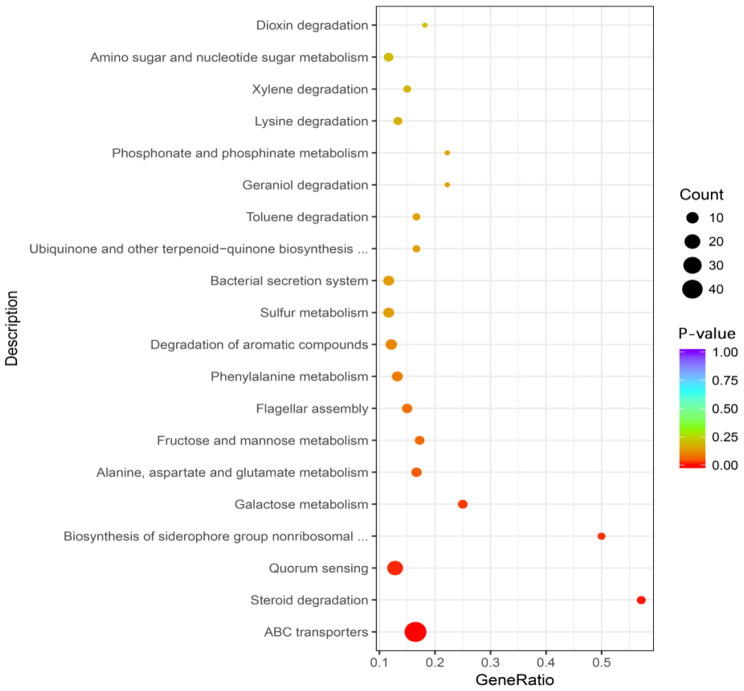
Enriched KEGG pathways among the DEGs in peanut RE-treated *Burkholderia pyrrocinia* P10. The KEGG enrichment analysis of the DEGs was performed using KOBAS. The degree of the KEGG enrichment was determined according to the Rich factor, the p-value, and the number of genes assigned to the pathway. The Rich factor refers to the ratio of the number of DEGs in the pathway to the total number of annotated genes in the pathway. Increases in the Rich factor correspond to increases in the degree of enrichment. The p-value range was [0,1]; values close to 0 reflect a significant enrichment

The enriched pathways among the DEGs in strain P10 were identified using the KEGG database (Supplementary Table S2). A total of 412 genes (83.91% of all DEGs) were assigned to 86 KEGG pathways, of which nine were differentially significant pathways (Fig. 3; Table 1). Notably, the up-regulated genes tended to be associated with ATP binding cassette (ABC) transporters, steroid degradation, quorum sensing (QS), biosynthesis of siderophore group nonribosomal peptides, and galactose metabolism. Most of the up-regulated genes were related to ABC transporters (Table 1). More specifically, 47 genes with expression levels that were up-regulated by 1.01- to 5.83-times were associated with the transport of minerals and organic ions, oligosaccharides, monosaccharides, amino acids, peptides, iron-siderophores, and ATP binding cassette subfamily C (ABCC) subfamily members. In some cases, the transcription of an entire gene cluster was observed, including the *ssuA-C-B* genes responsible for alkanesulfonate transport, *afuA-B-C* genes (Fe^3+^ transport), *proX-W-V* genes (glycine betaine/proline transport), and *araF-H-G* genes (L-arabinose transport). Additionally, the RE treatment of strain P10 up-regulated the expression of 19 genes involved in QS and four genes contributing to steroid degradation (i.e., conversion of androsta-1,4-diene-3,17-dione to 3-[3aS, 4 S, 7aS)-7a-methyl-1,5-dioxo-octahydro- 1 H-inden-4-yl]propanoyl-CoA (HIP-CoA)). The expression levels of three genes involved in the biosynthesis of siderophore group nonribosomal peptides and five genes related to galactose metabolism were also up-regulated. In contrast, several metabolic pathways were enriched among the down-regulated genes, namely flagellar assembly, two-component system, bacterial chemotaxis, and beta-lactam resistance. Six flagellar assembly-related genes were down-regulated, as were a gene encoding methyl-accepting chemotaxis protein I, which influences bacterial chemotaxis, and genes encoding the multidrug efflux system proteins MexX and MexY, which affect the two-component system and beta-lactam resistance.

**Table 1:** Enriched KEGG pathways among the DEGs in peanut RE-treated *Burkholderia pyrrocinia* P10

KEGG pathway	Number of DEGs	Number of all genes	DEGs ratio (%)	P*-*value	Regulated
ABC transporters	47	285	16.49	4.23E-08	Up
Steroid degradation	4	7	57.14	0.003330904	Up
Quorum sensing	19	156	12.18	0.011131881	Up
Biosynthesis of siderophore group nonribosomal peptides	3	6	50.00	0.014821194	Up
Galactose metabolism	5	20	25.00	0.016623881	Up
Flagellar assembly	6	40	15.00	1.90E-08	Down
Two-component system	5	145	3.45	0.000280868	Down
Bacterial chemotaxis	2	41	4.88	0.012845786	Down
beta-Lactam resistance	2	46	4.35	0.015850383	Down

### Effects of the peanut RE on the carbohydrate metabolism, transport, and energy production in strain P10

The peanut RE treatment induced the expression of multiple genes mediating carbohydrate transport and metabolism and fatty acid metabolism in strain P10 (Table 2). In terms of carbohydrate transport, the expression levels of genes encoding transporters of oligosaccharides (e.g., maltose, galactose oligomer, sorbitol, mannitol, and trehalose) and monosaccharides (e.g., ribose, xylose, and arabinose) were significantly up-regulated, thereby increasing the nutrients available to strain P10. The analysis of the DEGs related to carbohydrate metabolism suggested carbohydrate decomposition and energy production were accelerated and the reducing power increased in strain P10 following the peanut RE treatment. These changes resulted in the production of sufficient raw materials for the synthesis of cell components. Specifically, the expression of genes encoding beta-galactosidase (*bgaB*), aldose 1-epimerase (*galM*), 2-dehydro-3-deoxy-phosphogalactonate aldolase (*dgoA*), fructokinase (*scrK*), and mannose-6-phosphate isomerase (*algA*) was up-regulated, which enhanced the catabolism of sugars and the production of 3-phospho-glyceraldehyde. In addition, the expression levels of the 4-hydroxy-2-oxovalerate/4-hydroxy-2-oxohexanoate aldolase gene *dmpG* and the D-malate dehydrogenase gene *ywkA* were up-regulated, resulting in accelerated pyruvate production. The up-regulated expression of hydroxymethylglutaryl-CoA lyase-encoding gene *hmgL* increased acetyl-CoA production, leading to an increase in the raw materials necessary for the tricarboxylic acid (TCA) cycle. Moreover, the expression of the cytochrome O ubiquinol oxidase-encoding gene *cyoD* was up-regulated to accelerate energy production. The up-regulated expression of genes encoding xylose isomerase (*xylA*), xylulokinase (*xylB*), and D-arabinitol 4-dehydrogenase (*dalD*) increased the reducing power; most of these genes encode key enzymes in the sugar metabolic network. Among the DEGs related to the fatty acid synthesis and degradation pathway, the up-regulation of the long-chain acyl-CoA synthetase gene *fadD* accelerated the decomposition of fatty acids.

**Table 2:** Genes affected carbohydrate metabolism and transport in peanut RE-treated *Burkholderia pyrrocinia* P10

Gene ID & Gene name	Coding product	Log_2_FC	Gene ID & Gene name	Coding product	Log_2_FC
*ABD05_RS08670* *dgoK*	2-Dehydro−3-deoxy-galactono-kinase	1.37	*ABD05_RS08675* *dgoA*	2-Dehydro-3-deoxy-phosphogalactonate aldolase	3.82
*ABD05_RS19805* *dgoD*	Galactonate dehydratase	1.72	*ABD05_RS33495* *xylA*	Xylose isomerase	1.75
*ABD05_RS23380* *dalD*	D-arabinitol 4-dehydrogenase	1.43	*ABD05_RS03030* *scrK*	Fructokinase	2.05
*ABD05_RS20750*	L-fruconolactonase	2.09	*ABD05_RS33490* *xylB*	Xylulokinase	2.06
*ABD05_RS12485* *rffE*	UDP-N-acetylgluco-samine 2-epimerase	2.73	*ABD05_RS04160* *nagA*	N-acetylglucosamine−6- phosphate deacetylase	−1.31
*ABD05_RS34190* *rfbD*	dTDP−4-dehydro- rhamnose reductase	1.11	*ABD05_RS18190* *ywkA*	D-malate dehydrogenase	1.56
*ABD05_RS27705* *echA15*	Enoyl-CoA hydratase	1.55	*ABD05_RS10755* *fdxH*	Formate dehydrogenase	1.05
*ABD05_RS25365* *ackA*	Acetate kinase	1.45	*ABD05_RS29405*	Gluconate 2-dehydrogenase	2.28
*ABD05_RS17755* *catB*	Muconate cycloisomerase	1.69	*ABD05_RS21650* *araG*	L-arabinose transport system ATP-binding protein	1.65
*ABD05_RS27815* *dmpE*	2-Oxopent−4-enoate/ cis−2-oxohex−4-enoate hydratase	3.33	*ABD05_RS27805* *dmpG*	4- Hydroxy−2-oxo- valerate/4-Hydroxy−2-oxohexanoate aldolase	1.39
*ABD05_RS23950* *fdhA*	Glutathione-independent formaldehyde dehydrogenase	1.32	*ABD05_RS00255* *cyoD*	Cytochrome o ubiquinol oxidase	1.25
*ABD05_RS21580* *malK*	Multiple sugar transport system ATP-binding protein	1.89	*ABD05_RS24180* *rbsC*	Ribose transport system permease protein	1.15
*ABD05_RS21645* *araF*	L-arabinose transport system substrate-binding protein	1.15	*ABD05_RS21655* *araH*	L-arabinose transport system permease protein	2.24
*ABD05_RS33475* *xylH*	D-xylose transport system permease protein	2.20	*ABD05_RS33480* *xylG*	D-xylose transport system ATP-binding protein	1.76
*ABD05_RS31300* *fabH*	3- Oxoacyl-[acyl- carrier-protein] synthase III	3.04	*ABD05_RS30960* *fadD*	Long-chain acyl-CoA synthetase	1.61

Note: FC is the abbreviations of Foldchange, the same below

### Effects of the peanut RE on the metabolism and transport of amino acids and nutrients in strain P10

Amino acid transport and metabolism in strain P10 were also induced by the peanut RE treatment. The genes encoding the transporters of proline, lysine, arginine, histidine, cysteine, branched amino acids, methionine, glutathione, and other amino acids had expression levels that were up-regulated by 1.07- to 4.67-times. The expression levels of multiple genes involved in amino acid metabolism, degradation, and biosynthesis pathways were also up-regulated (Table 3). For example, the up-regulated expression of genes encoding the enoyl-CoA hydratase (*paaF*) and the acetyl-CoA acyltransferase (*pcaF*) resulted in the increased production of acetyl-CoA. The up-regulation of genes encoding 5-oxoprolinase (*pxpA*) and glutathione S-transferase (*gstB*) increased the production of glutamate and glutathione. In the arginine biosynthesis pathway, the expression of the acetylornithine deacetylase gene *argE* was up-regulated, leading to increased citrulline and ornithine synthesis. The expression of the GMC oxidoreductase family protein-encoding gene *betA*, which catalyzes the formation of betaine aldehyde, increased by 2.60-times.

**Table 3:** Genes affected other nutriments metabolism and transport in peanut RE-treated *Burkholderia pyrrocinia* P10

Gene ID & Gene name	Coding product	Log_2_FC	Gene ID & Gene name	Coding product	Log_2_FC
*ABD05_RS23040* *hppD*	4- Hydroxyphenyl- pyruvate dioxygenase	1.13	*ABD05_RS27705* *paaF*	Enoyl-CoA hydratase (PaaF)	1.55
*ABD05_RS25490* *asnB*	Asparagine synthase	1.31	*ABD05_RS25685* *pyrB*	Aspartate carbamoyltransferase	−1.02
*ABD05_RS12450* *glmS*	Glutamine–fructose−6- phosphate transaminase	1.39	*ABD05_RS29345* *hpaF*	5- Carboxymethyl- 2-hydroxymuconate isomerase	−1.07
*ABD05_RS18950* *pxpA*	5-oxoprolinase	1.02	*ABD05_RS18870* *gstB*	Glutathione S-transferase	1.19
*ABD05_RS19810* *betA*	GMC oxidoreductase family protein	2.60	*ABD05_RS18560* *soxD*	FAD binding domain protein	2.52
*ABD05_RS27720* *pcaF*	Acetyl-CoA acyltransferase	2.72	*ABD05_RS22400* *hmgL*	Hydroxymethylglutaryl-CoA lyase	1.59
*ABD05_RS16435* *mmsB*	3-Hydroxyisobutyrate dehydrogenase	1.69	*ABD05_RS31940* *argE*	Acetylornithine deacetylase	1.32
*ABD05_RS32735* *uca*	Urea carboxylase	2.64	*ABD05_RS24815* *aroK*	Shikimate kinase	2.37
*ABD05_RS14910* *aroF*	3- Deoxy−7-phospho- heptulonate synthase	3.17	*ABD05_RS34210* *dapA*	4- Hydroxy-tetrahydro- dipicolinate synthase	1.52
*ABD05_RS34330* *potI*	Putrescine transport system permease protein	3.11	*ABD05_RS30720* *argT*	Lysine/arginine/ornithine transport system substrate-binding protein	2.98
*ABD05_RS05015* *porG*	Putrescine transport system ATP-binding protein	4.67	*ABD05_RS23590* *proX*	Glycine betaine/proline transport system substrate-binding protein	2.13
*ABD05_RS19845* *proW*	Glycine betaine/proline transport system permease protein	1.96	*ABD05_RS23575* *proV*	Glycine betaine/proline transport system ATP-binding protein	2.26
*ABD05_RS18475* *hisJ*	Histidine transport system substrate-binding protein	3.93	*ABD05_RS10140* *tcyB*	L-cystine transport system permease protein	2.42
*ABD05_RS19175* *livK*	Branched-chain amino acid transport system substrate-binding protein	1.07	*ABD05_RS12875* *livM*	Branched-chain amino acid transport system permease protein	1.20
*ABD05_RS12885* *livF*	Branched-chain amino acid transport system ATP-binding protein	1.25	*ABD05_RS22925* *metN*	D-methionine transport system ATP-binding protein	1.63
*ABD05_RS22075* *oppB*	Oligopeptide transport system permease protein	1.11	*ABD05_RS14560* *gsiD*	Glutathione transport system permease protein	1.47
*ABD05_RS27435* *narK*	Nitrate/nitrite transporter Nrt	1.99	*ABD05_RS27440* *nirBD*	Nitrite reductase	1.56
*ABD05_RS10140* *urtD*	Urea transport system ATP-binding protein	2.42	*ABD05_RS10145* *urtC*	Urea transport system permease protein	1.44
*ABD05_RS00860* *cysCDN*	Sulfate adenylyltransferase Adenylylsulfate kinase	1.32	*ABD05_RS27450* *cysI*	Sulfite reductase	1.02
*ABD05_RS16555* *ssuC*	Sulfonate transport system permease protein	2.28	*ABD05_RS16565* *ssuB*	Sulfonate transport system ATP-binding protein	3.82
*ABD05_RS29265* *ssuA*	Sulfonate transport system substrate-binding protein	5.83	*ABD05_RS04460* *tauB*	Taurine transport system substrate-binding protein	2.32

The expression levels of the nitrogen transport and metabolism genes encoding the urea transport system proteins UrtC and UrtD, the nitrate/nitrite transporter Nrt, which transports nitrate/nitrite into cells, and the nitrite reductase NirBD, which converts nitrate to ammonia, were up-regulated. In terms of sulfur transport, the expression levels of three genes encoding the alkanesulfonate transporters SsuA (substrate-binding protein), SsuC (permease protein), and SsuB (ATP-binding protein) were up-regulated by 1.39- to 5.83-fold. Similarly, the expression of the taurine transport system ATP-binding protein-encoding gene *tauB* was also up-regulated (2.32-fold). Additionally, the expression of seven sulfur metabolism-related genes was also induced, including genes encoding the sulfate adenylyltransferase CysND, the adenylylsulfate kinase CysC, and the sulfite reductase CysJI.

### Effects of the peanut RE on strain P10 biofilm formation, competition, and colonization

The ability of PGPR to colonize the rhizosphere is a key factor influencing their plant growth-promoting effects. The 2-h incubation in a culture containing the peanut RE altered the expression of some genes affecting strain P10 motility and chemotaxis (Table 4). Of the flagellar assembly-related genes, the expression levels of *fliD* (flagellar cap), *fliC* (flagellin), *flgK* (hook–filament junction), and *flgN* (flagellar biosynthesis) were down-regulated, with detrimental consequences for the synthesis of flagella. In addition, the expression levels of the genes encoding the chemotaxis protein MotB and the methyl-accepting chemotaxis protein MCP were down-regulated.

**Table 4:** Genes affected the mobility, chemotaxis, and biofilm of peanut RE-treated *Burkholderia pyrrocinia* P10

Gene ID & Gene name	Coding product	Log_2_FC	Gene ID & Gene name	Coding product	Log_2_FC
*ABD05_RS06825* *fliD*	Filament cap	−1.15	*ABD05_RS05110* *flgK*	Hook-filament junction	−1.37
*ABD05_RS06830* *filC*	Flagellin	-1.51	*ABD05_RS29500* *algA*	Mannose−1-phospho- guanyltransferase/ mannose−6-phospho- isomerase	2.37
*ABD05_RS05175* *flgN*	Flagellar biosynthesis protein	−1.56	*ABD05_RS06885* *motB*	Chemotaxis protein	−1.00
*ABD05_RS26665* *mcp*	Methyl-accepting chemotaxis protein	−1.37	*ABD05_RS20305* *bcl*	Mannose-binding lectin	4.94
*ABD05_RS24095* *zmpB*	Zinc metalloprotease	2.45	*ABD05_RS22075* *oppA*	Oligopeptide transport system substrate-binding protein	1.11
*ABD05_RS05815* *plcA*	Phospholipase C	1.49	*ABD05_RS14910* *phzC*	3-Deoxy−7-phosphoheptulonate synthase	3.17
*ABD05_RS18865* *vgrG*	Type VI secretion system secreted protein VgrG	1.44	*ABD05_RS17850* *clpV*	Type VI secretion system protein ClpV	1.97
*ABD05_RS25480* *yscT*	Type III secretion protein T (YscT)	2.82	*ABD05_RS25505* *yscR*	Type III secretion protein R (YscR)	3.65
*ABD05_RS25425* *yscV*	Type III secretion protein V YscV	1.67	*ABD05_RS25465* *yscL*	Type III secretion protein L (YscL)	5.59
*ABD05_RS08705* *galM*	Aldose 1-epimerase	1.18	*ABD05_RS21575* *bgaB*	Beta-galactosidase	2.62

The peanut RE treatment also modulated the expression of genes associated with strain P10 adhesion and biofilm formation. In the pathways mediating the metabolism of glucose and mannose, amino sugars, and ribose, the expression of the mannose-1-phosphoguanyltransferase gene *algA*, which encodes the enzyme that converts mannose-1-phosphate to GDP-mannose, was up-regulated. The generated GDP-mannose is an important exopolysaccharide (EPS) constituent and the main component of biofilms. In the galactose metabolic pathway, the up-regulated expression of *bgaB* and *galM* lead to increased α-D-galactose production. The expression levels of the genes encoding the inner member protein YscT, YscR, YscV, and the ATPase-associated protein YscL subunits of the Type III secretion system and the secreted protein VgrG and secretion ATPase ClpV components of the Type VI secretion system were up-regulated. Moreover, the expression of the GspK-encoding gene in the Type II secretion system was up-regulated by 4.52-times. In the QS system, the up-regulated genes included genes encoding a mannose-binding lectin (Bcl; up-regulated 4.94-times), a zinc metalloprotease (ZmpB), an oligopeptide transport system substrate-binding protein (Opp), a phospholipase C (PlcA), and a 3-deoxy-7-phosphoheptulonate synthase (PhzC). These proteins influence biofilm formation and virulence. Additionally, the up-regulated expression of 12 genes involved in the biosynthesis of antibiotics, such validamycin, streptomycin, and enediyne antibiotics, as well as polyketide sugar units provided strain P10 with favorable conditions for increasing its competitiveness and biofilm-forming abilities.

### Effects of the peanut RE on the plant growth-promoting activities of strain P10

The peanut RE treatment also improved the growth-promoting activities of strain P10. Of the genes contributing to the biosynthesis of siderophore group nonribosomal peptides, the expression levels of the L-cysteine-ligase gene *pchE*, the salicylate-ligase gene *pchD*, and the isochorismate synthase gene *pchA* were significantly up-regulated (Table 5). The genes encoding the Fe^3+^ transport system substrate-binding protein AfuA, the permease AfuB, the ATP-binding protein AfuC, and the ferric hydroxamate transport system permease FhuB also had up-regulated expression levels, indicating that siderophore synthesis in strain P10 was enhanced and the uptake and transport capacity of Fe^3+^ increased in response to the peanut RE treatment. In the tryptophan metabolic pathway, the expression of *amiE*, which encodes an amidase that catalyzes the conversion of indole-3-acetamide to indoleacetate, was up-regulated, which resulted in increased IAA production. In addition, the expression of genes encoding an isocitrate lyase (*aceA*), a glutarate-/succinate-semialdehyde dehydrogenase (*gabD*), an argininosuccinate lyase (*argH*), a formyltetrahydrofolate deformylase (*purU*), and a hippurate hydrolase (*hipO*) increased. The resulting increased production of organic acids may have promoted phosphorus solubilization-related activities.

**Table 5:** Genes influencing the plant growth-promoting activities of peanut RE-treated *Burkholderia pyrrocinia* P10

Gene ID & Gene name	Coding product	Log_2_FC	Gene ID & Gene name	Coding product	Log_2_FC
*ABD05_RS14930* *pchE*	L-cysteine—[L-cysteinyl-carrier protein] ligase	4.39	*ABD05_RS14935* *pchD*	Salicylate—[aryl-carrier protein] ligase	2.42
*ABD05_RS14940* *pchA*	Isochorismate synthase	3.43	*ABD05_RS34310* *amiE*	Amidase	1.40
*ABD05_RS11775* *afuA*	Iron(III) transport system substrate-binding protein	1.12	*ABD05_RS09890* *afuB*	Iron(III) transport system permease protein	1.68
*ABD05_RS11765* *afuC*	Iron(III) transport system ATP-binding protein	1.01	*ABD05_RS14000* *fhuB*	Ferric hydroxamate transport system permease protein	1.66
*ABD05_RS24050* *purU*	Formyltetrahydrofolate deformylase	3.24	*ABD05_RS00140* *aceA*	Isocitrate lyase	1.30
*ABD05_RS10755* *fdxH*	Formate dehydrogenase	1.05	*ABD05_RS30705* *argH*	Argininosuccinate lyase	1.47
*ABD05_RS19015* *hipO*	Hippurate hydrolase	2.22	*ABD05_RS19795* *gabD*	Glutarate-/succinate- semialdehyde dehydrogenase	1.89
*ABD05_RS23060* *phnW*	2-aminoethylphosphonate-pyruvate transaminase	2.15			

### Quantitative real-time polymerase chain reaction (Q-RT-PCR) analysis of selected DEGs

Twelve genes were selected to evaluate the reliability of the strain P10 transcriptome data. Gene-specific primers were designed for the Q-RT-PCR analysis. Although there were some differences in the fold-changes of several significant DEGs between the Q-RT-PCR and RNA-seq analyses, the general trends were consistent, suggesting that the RNA-seq data were reliable (Supplementary Fig. 1).

### Analysis of peanut RE components

The peanut RE was mainly composed of organic acids and amino acids, but it also contained sugars, alcohols, fatty acids, sugar alcohols, sugar acids, and other components (Supplementary Table S3). The detected compounds included low-molecular-weight organic acids, such as malic acid, lactic acid, succinic acid, pyruvic acid, oxalic acid, and citric acid, which were present at relatively high concentrations. Various amino acids were also detected, including alanine, glycine, proline, valine, phenylalanine, isoleucine, tyrosine, methionine, threonine, glutamic acid, serine, lysine, asparagine, glutamine, and aspartic acid. Xylose, allose, lyxose, and ribose were the most prominent sugars in the peanut RE, which also contained fatty acids (e.g., palmitic acid, stearic acid, myristic acid, oleic acid, and palmitoleic acid), alcohols (e.g., 4-hydroxyphenylethanol, myo-inositol, and phytol), sugar alcohols (e.g., threitol, xylitol, sorbitol, and arabitol), sugar acids (e.g., galactonic acid, gluconic acid, and threonic acid), and some other components (e.g., indole-3-acetamide and urea). Accordingly, low-molecular-weight organic acids were the major carbon-containing compounds in the peanut RE.

### Effects of specific organic acids and amino acids on the biofilm formation and IAA secretion of strain P10

**Fig. 4:**
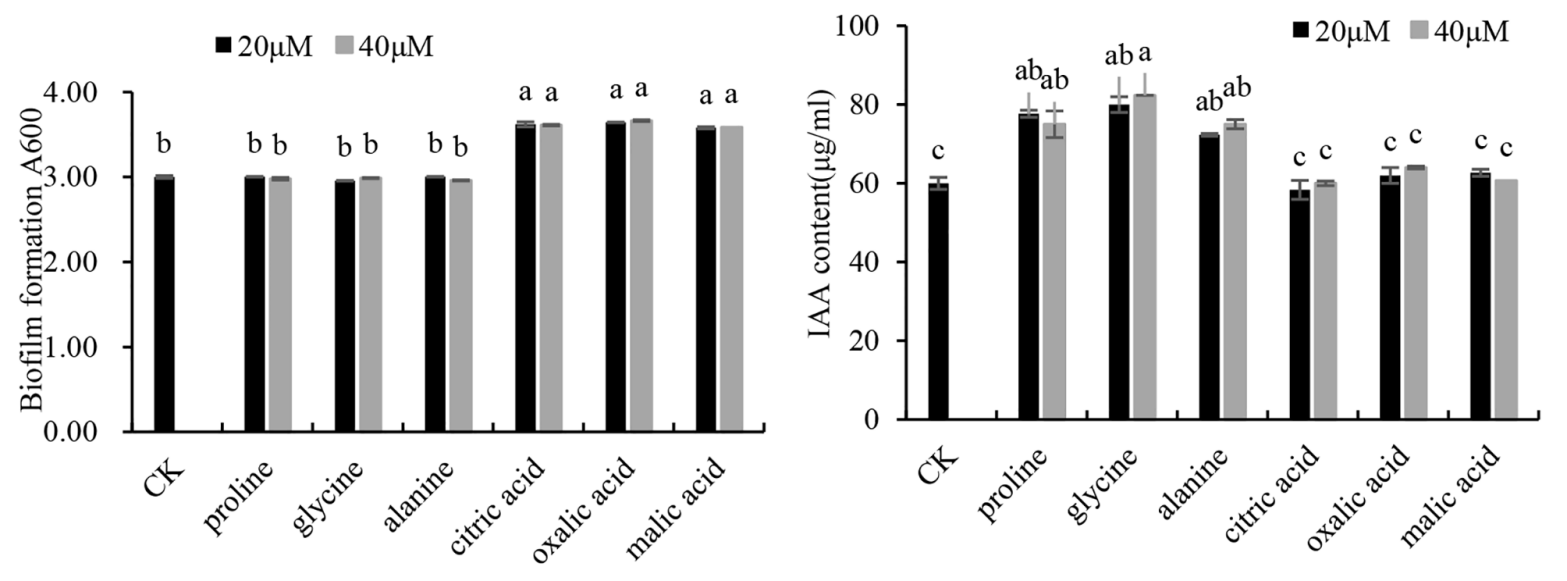
Effects of RE components on *Burkholderia pyrrocinia* P10 biofilm formation and IAA secretion. The A_600_ value reflects the biofilm formation. The IAA content was measured according to the Salkowski method. Bars indicate the standard errors of the means from three replicates. Columns with different letters are statistically different according to the Duncan test (*P* < 0.05)

To identify the peanut RE components that promote the growth of strain P10 and enhance its plant growth-promoting effects, three organic acids (malic acid, oxalic acid, and citric acid) and three amino acids (alanine, glycine, and proline) were selected on the basis of the chemical analysis of the peanut RE and the related published literature for further analyses. The formation of the strain P10 biofilm was significantly induced by the three organic acids (*P* < 0.05; Fig. 4), but was unaffected by the three amino acids. In contrast, the secretion of IAA by strain P10 increased in response to the three amino acids, but was not affected by the three organic acids.

## Discussion

Plant REs are important for the communication between the root system and rhizospheric microorganisms and the regulation of root development. Plant–PGPR interactions are primarily mediated by REs, which serve as the main nutrient source for PGPR, while also promoting PGPR adhesion, colonization, and biofilm formation [6]. The REs of several plants typically comprise many small molecular compounds, including amino acids, organic acids, fatty acids, sugars, and secondary metabolites [19–21]. In accordance with the findings of these earlier investigations, in the current study, we detected organic acids, amino acids, sugars, alcohols, fatty acids, sugar alcohols, and sugar acids in the peanut RE. The transcriptome analysis indicated the peanut RE accelerated nutrient metabolism and transport in *B. pyrrocinia* P10, but it also promoted the growth and reproduction of this strain. Furthermore, it up-regulated the expression of the genes associated with biofilm formation, phosphorus solubilization, IAA production, and siderophore secretion, thereby increasing the plant growth-promoting effects of strain P10.

### Peanut RE activated the metabolism and transport of carbon, nitrogen and sulfur of P10 strain during the early interaction

The expression levels of some genes involved in the metabolism or transport of carbohydrates or amino acids were altered in response to the peanut RE treatment. The RNA-seq analysis of *B. pyrrocinia* P10 revealed the up-regulated expression of various genes related to carbohydrate metabolism, including genes involved in galactose metabolism, fructose and mannose metabolism, amino sugar and nucleotide sugar metabolism, and pentose and glucuronate interconversions (Table 2). We also detected galactose, xylose, sucrose, and fructose in the peanut RE (Supplementary Table 3). Accordingly, the expression of some ABC transporter-related genes was up-regulated, including genes encoding multiple sugar transport system ATP-binding proteins. The expression levels of genes encoding proteins in the ribose transport system (RbsC and RbsA), the L-arabinose transport system (AraFHG), and the D-xylose transport system (XylH and XylG) were also up-regulated. Similar findings were reported for *Pseudomonas fluorescens* treated with the *Brachypodium distachyon* RE; these changes may contribute to the differential affinity of *Pseudomonas* for host plants and/or determine which strains can flourish in response to root growth and changes in environmental conditions [9]. The peanut RE also stimulated the expression of genes related to the transport of multiple amino acids, including proline, lysine, histidine, branched-chain amino acids, and glutathione. The expression levels of most of the genes involved in the metabolism of tryptophan, arginine, proline, alanine, aspartate, and glutamate were up-regulated by the peanut RE treatment (Table 3). These results along with the strain P10 growth curve (Fig. 1) suggest that the peanut RE is a source of nutrients required for strain P10 growth. More specifically, the compounds in the peanut RE induce the expression of functional genes encoding proteins responsible for transporting and metabolizing certain substances in strain P10, thereby promoting the growth and reproduction of the strain. Similar results were obtained in an earlier study that examined the effects of pepper on *Paenibacillus polymyxa* SC2 [22]. In addition to nutrient availability, bacterial survival in the rhizosphere depends on the ability of the microbe to tolerate environmental stresses. In the current study, the expression of the glutathione S-transferase-encoding gene was up-regulated, resulting in increased glutathione levels. And the induced expression of the GMC oxidoreductase family protein-encoding gene *betA* led to the increased production of betaine, which protects against osmotic stress [23]. The expression levels of genes encoding the transporters of nitrite/nitrate and urea, as well as assimilatory nitrate reduction were also up-regulated. Similar findings were reported for the rhizospheric interaction between *Herbaspirillum seropedicae* and maize roots [24].

Interestingly, the expression of *ssuACB*, which are involved in sulfur transport, and *tauB*, which contributes to taurine transport, was activated by the peanut RE treatment. These transporter genes enable the use of alkanesulfonates and taurine (2-aminoethanesulfonate) as sulfur sources. Although the preferred sulfur source for *B. pyrrocinia* is unknown, these findings combined with the fact sulfur-containing amino acids were not detected in the peanut RE imply that strain P10 may activate sulfur transport as sulfur source. Accordingly, the expression of the gene encoding the sulfite reductase CysJI was activated to increase the production of sulfide, which is the precursor of cysteine. The up-regulated expression of genes encoding the sulfate adenylyltransferase CysND and the adenylylsulfate kinase CysC lead to the accumulation of adenylyl sulfate and phosphoadenylyl sulfate. These changes during the early stage of PGPR–plant interactions have not been thoroughly characterized. Nevertheless, the link between sulfur metabolism and virulence has been reported for several bacterial pathogens. For example, it affects the long-term adaptation of *B. cenocepacia* during the colonization of the cystic fibrosis patients [25]. Pimenta et al. observed that the expression levels of genes involved in sulfur metabolism are up-regulated by REs. These molecular changes reportedly occur during the early stage of the adhesion of pathogenic *B. cenocepacia* K56-2 to the host cell giant plasma membrane vesicles (GPMVs) derived from live bronchial epithelial cells [26]. The assimilation of sulfur from inorganic sulfate and other sources seems to be a rapid response of *B. cenocepacia* to the initial interactions with the host [27]. These findings may help to explain the observed changes in *B. pyrrocinia* P10 after a 2-h exposure to the peanut RE.

### Peanut RE induced the biofilm formation and colonization of P10 strain during the early interaction

Most PGPR can effectively colonize the rhizosphere and form biofilms because of plant–microbe interactions [28]. As the main component of biofilms, EPS is positively correlated with the ability of PGPR to colonize the rhizosphere [29]. Although the expression of genes directly related to biofilm formation (e.g., *epsA–O*) did not differ significantly from the control (i.e., not treated with the peanut RE), the biofilm formation induced by the peanut RE may be attributed to the activation of metabolism-related genes that induce growth and cell proliferation (Fig. 1). This possibility is supported by the positive effects of three organic acids on biofilm formation (Fig. 4). These observations are consistent with the results of an earlier study on *Bacillus amyloliquefaciens* strain SQR9 responses to maize REs [30]. In many bacterial species, sugar nucleotides, such as UDP-galactose, are essential for EPS biosynthesis. Moreover, galactose is critical for the synthesis of various EPSs;. *Pseudomonas aeruginosa* secretes alginate to facilitate the formation of thick highly structured biofilms, the biosynthesis of which involves a single 12-gene operon (e.g., *algA*, *algD*, and *algK*) and *algC*. In the present study, the expression levels of a gene encoding β-galactosidase *bgaB*, which converts galactan to D-galactose, and a gene encoding aldose 1-epimerase *galM*, which catalyzes the production of α-D-galactose, were up-regulated by the peanut RE. In addition, AlgA in strain P10 is a bifunctional enzyme (i.e., phosphomannose isomerase–guanosine 5′-diphospho-D-mannose pyrophosphorylase activities). All of these enzymatic reactions result in the accumulation of substantial amounts of raw materials needed for biofilm formation.

Biofilm maturation mainly depends on the accumulation of the extracellular matrix and the QS signal [31]. Quorum sensing has critical regulatory effects on bacterial activities and characteristics, including biofilm formation, antibiotic resistance, and bioluminescence [32, 33]. In our study, 19 QS-related DEGs were up-regulated (1.06- to 4.94-fold). These DEGs included a gene encoding the 3-deoxy-7-phosphoheptulonate synthase PhzC, which catalyzes the production of phenazine, and a gene encoding the mannose-binding lectin Bcl. The up-regulated expression of these genes enable strain P10 to perceive environmental changes, while also promoting biofilm formation. The expression of the QS-related genes in *P. polymyxa* SC2 is also up-regulated following an interaction with pepper [22]. Recent studies showed that the secretion system helps facilitate host–microorganism interactions. For example, the Type III protein secretion system (T3SS) is required for the secretion or translocation of effector proteins; the T3SS in *P. aeruginosa* is induced in the sugar beet rhizosphere [34]. The Type VI secretion system (T6SS) is commonly used to export proteins and is involved in essential processes, especially in pathogenic bacteria, including bacterial interactions, biofilm formation, and the competition for essential nutrients [35–37]. The valine–glycine repeat protein G (VgrG) is a virulence factor in many Gram negative bacilli [38]. Klonowska et al. were the first to describe the inductive effects of *M. pudica* REs on the expression of T6SS-encoding genes in *B. phymatum* STM815 [39]. In our study, the expression levels of genes encoding the inner membrane protein YscRTUV and the ATPase-associated protein YscL of T3SS, VgrG and ClpV of T6SS, and GspK of T2SS were up-regulated by 1.44- to 5.59-fold by the peanut RE treatment. Thus, the peanut RE can induce *B. pyrrocinia* P10 responses to environmental stimuli. In addition, the activation of T6SS may provide PGPR with a competitive advantage over their rhizobial competitors [40, 41].

We observed the down-regulated expression (1.00- to 1.51-fold) of flagellar assembly-related genes (*fliC*, *fliD*, *flgK*, *flgN*, and *motB*) and chemotaxis-related gene *mcp* after a 2-h treatment with the peanut RE. In the structure of flagella, both FliC and FliD form the helical filament, whereas FlgK is the component of hook filament junction and MotB is the stator of basal body [42, 43]. Chemotaxis and flagella-driven motility, which are crucial for the bacterial colonization of roots, are induced by REs in many rhizospheric bacteria [44, 45]. In *P. polymyxa* SC2, a 20-h exposure to the stimulatory effects of pepper leads to the up-regulated expression of chemotaxis genes (e.g., *cheA*, *cheY*, *cheD*, and *cheC*) and *fliM* and *fliN*, which encode flagellar motor switch proteins [22]. At 24 h post-inoculation with maize REs, the expression levels of genes involved in cell motility and chemotaxis are up-regulated in *B. amyloliquefaciens* SQR9 [30]. Interestingly, Xie et al. examined the expression of a malate dehydrogenase gene (*ywkA*), a UDP-glucose-4-epimerase gene (*galE*), and an L-arabinose isomerase gene (*araA*), which are indicators of carbohydrate degradation, and observed that *B. subtilis* OKB105 quickly colonizes rice seedling roots and begins to use plant carbohydrates after 2 h, but the expression levels of genes associated with chemotaxis and motility are down-regulated to conserve energy [12]. In the current study, the *ywkA* expression level in strain P10 was up-regulated by 1.56-fold (Table 2). Moreover, a 30-min incubation with GPMVs suppresses the expression of *B. cenocepacia* genes involved in the chemotaxis signaling pathway, whereas the *fliC*, *flgK*, *motA*, and *mcp* expression levels are down-regulated upon adhesion [26]. Plant–microbe interactions are highly complex. The ability to sense/recognize specific signals and activate RE-related chemotaxis varies among rhizobia and is influenced by the duration of the plant host–microbe interaction. Therefore, we speculate that within 2 h of an exposure to the peanut RE, which coincides with the early logarithmic growth phase, *B. pyrrocinia* P10 begins to use compounds in the RE as nutrients and starts to adapt to the changing environment. These modifications lay the foundation for the subsequent biofilm formation and colonization. In addition to the changes in the expression of the genes related to EPS biosynthesis, QS, and secretion systems, the up-regulated expression of *xylA* and *nirBD* also lead to biofilm formation. These genes are reportedly essential for the colonization of maize and beet by *H. seropedicae* and *Pseudomonas fluorescens* [24, 46].

### Peanut RE improved the plant growth-promoting characteristics of P10 strain during the early interaction

Our transcriptome data revealed that the growth-promoting effects of strain P10 were obviously induced by the peanut RE. Iron is an essential nutrient for bacterial growth. Some PGPR secrete siderophores, which are low-molecular-weight compounds with a high affinity for iron in the environment. When iron is limited, microbial siderophores also provide plants with iron to enhance growth [47]. In the present study, we observed that the peanut RE treatment activated a series of genes involved in iron and ferric hydroxamate transport, including *afuA/B/C* and *fhuB*, and genes related to siderophore biosynthesis (e.g., *pchE*, *pchD*, and *pchA*). Similarly, after an interaction with barley roots, the expression levels of a siderophore gene cluster are up-regulated in *Paenibacillus* sp. strains [48]. In *B. amyloliquefaciens*, some genes involved in iron transport and siderophore biosynthesis are also induced by maize REs [30]. Previous research confirmed that iron has important roles related to the biofilm formation of diverse bacteria, including *Staphylococcus aureus*, *P. aeruginosa*, *E. coli*, and *Vibrio cholerae* [49, 50]. The up-regulated expression of these iron transporter genes may promote the formation of strain P10 biofilms. IAA is a primary plant hormone that regulates growth, could be synthesized by amidase in the indole-3-acetamide (IAM) pathway [51]. In this study, the *amiE* expression level in strain P10 was up-regulated after a 2-h incubation, implying the peanut RE promoted auxin biosynthesis, possibly because it contained tryptophan, which is a precursor for IAA synthesis. In *P. polymyxa* YC0136, the expression of *ilvB* in the IAA biosynthesis-related indole-3-pyruvate pathway is induced by tobacco REs [52]. In addition, tryptophan in REs stimulates the colonization of the rhizosphere by *Burkholderia phytofirmans* [53]. It is possible that increases in IAA secretion may enhance biofilm formation. A large proportion of the inorganic phosphates in fertilizers applied to the soil is rapidly immobilized so it is unavailable to plants. Certain rhizobacteria are able to solubilize insoluble or poorly soluble mineral phosphates because they produce phosphatases and organic acids [54]. Phosphonates are organophosphorus molecules that contain the highly stable C–P bond. The genes mediating phosphonate uptake and degradation in *E. coli* are present in a *phn* operon [55]. We detected the peanut RE-induced expression of some genes encoding enzymes that catalyze the production of succinic acid, fumaric acid, and formate, including *aceA*, *gabD*, *argH*, and *purU*. The expression of the 2-aminoethylphosphonate-pyruvate transaminase-encoding gene *phnW* was also induced, which likely increased the ability of strain P10 to solubilize phosphorus. Hence, the peanut RE clearly enhances the plant growth-promoting effects of strain P10. To the best of our knowledge, this is the first report describing the transcriptomic changes in *B. pyrrocinia* during the early interaction with the peanut RE. The data presented herein provide the basis for future investigations of the adaptive changes in PGPR during the interaction with plant hosts as well as the mechanism underlying the interaction.

In addition, to clarify the effects of peanut RE components on the growth, reproduction, and plant growth-promoting activities of strain P10, we selected three organic acids and three amino acids for an examination of biofilm formation and IAA secretion. In the peanut RE, malic acid, oxalic acid, and citric acid were the common low-molecular-weight organic acids. All three organic acids positively affected strain P10 biofilm formation, suggesting that the peanut RE can alter the expression of genes to enhance biofilm formation. Organic acids in *Limonium sinense* REs provide nutrients for *Bacillus flexus* growth, while also serving as signaling molecules for plant–rhizobacteria interactions [56]. Citric acid, malic acid, and oxalic acid induce the formation of biofilms and promote the colonization by different strains of *Xylella fastidiosa* and *Bacillus* sp. [57, 58]. Notably, some amino acids in REs may influence plant–PGPR interactions. For example, the chemotactic responses of *P. fluorescens*, *P. putida*, and *Sinorhizobium meliloti* to nonpolar neutral amino acids have been reported [59, 60]. Moreover, *P. protegens* is obviously attracted to neutral amino acids, but not to acidic or basic amino acids [61]. In the current study, three nonpolar neutral amino acids (proline, glycine, and alanine) did not affect biofilm formation, but they promoted the secretion of IAA by strain P10. These findings indicate that a specific RE component may be responsible for specific PGPR–plant interactions [62]. Therefore, further study of RE component of peanut on the growth promoting mechanisms of P10 strain, which of great significance to clearly clarify the interaction of P10-peanut.

## Conclusion

In this study, a transcriptome analysis of *B. pyrrocinia* P10 was performed following a peanut RE treatment. The peanut RE positively affected the growth of strain P10. The transcriptome analysis revealed that the peanut RE enhanced the expression of genes mediating the transport and metabolism of carbohydrates, amino acids, nitrogen, and sulfur. Although the expression of some flagellar assembly-related genes was down-regulated, the peanut RE had the opposite effect on the expression of genes related to EPS biosynthesis, QS, and the bacterial secretion system. Hence, strain P10 responds rapidly and positively to the peanut RE. Moreover, the peanut RE treatment activated siderophore biosynthesis, IAA production, and phosphorus solubilization, thereby enhancing the plant growth-promoting effects of strain P10. Furthermore, three organic acids in the peanut RE significantly induced strain P10 biofilm formation, whereas three amino acid components promoted the secretion of IAA by this strain. The findings of this study have further clarified the mechanism by which *B. pyrrocinia* promotes plant growth.

## Methods

### Bacterial strain and plants

*Burkholderia pyrrocinia* P10 was previously isolated from the rhizosphere soil of tea tree in the Kuankuoshui National Nature Reserve in Zunyi, China [63]. It is currently stored at the China Center for the Preservation of Typical Cultures (strain preservation number: CCTCC M 2019172) and our laboratory as frozen stocks (-80 °C) and routinely cultured in Luria-Bertani (LB) medium. Peanut seeds (*Arachis hypogaea* L.) were purchased from Huaxi Seed Company, Guizhou, China.

### Collection of the peanut RE

‘Qianhuasheng 5’ peanut seeds were surface-sterilized in a 20% H_2_O_2_ solution for 20 min. They were subsequently repeatedly rinsed with sterile water, soaked for 8 h, and then germinated on two layers of moistened sterile filter paper in Petri dishes during a 3-day incubation at 28 °C in a light incubator (16-h light/8-h dark photoperiod). Uniformly growing seedlings were transferred to pots (1 kg soil/pot) and then incubated in a greenhouse. After 30 days of growth, 40 seedlings were uprooted from the soil, after which the roots were gently washed four times with sterile double-distilled water to remove the adhering soil particles. Four seedlings were added to 100-mL flasks so that the roots were submerged in 50 mL sterile double-distilled water. The samples were incubated for 5 days at 28 °C in a light incubator. The RE solutions (2,000 mL) were collected from 40 seedlings and filtered through a 0.22 μm membrane (Millipore, Billerica, MA, USA). The filter-sterilized RE samples were lyophilized to a volume of 50 mL and stored at − 80 °C until they were analyzed.

### Analysis of the effects of different peanut RE concentrations on the growth of strain P10

Using LB liquid medium as the control, different peanut RE concentrations (0.5%, 1.0%, and 2.0%) were added to the LB medium for the subsequent treatments. First, strain P10 was grown on LB agar medium (overnight at 30 °C). A pure culture of this strain was used to inoculate the corresponding liquid medium (adjusted initial OD_600_ = 0.1), which was then incubated at 30 °C for 14 h on a rotary shaker (150 rpm) to analyze the growth of strain P10. Each treatment was performed using three replicates.

### Analysis of the strain P10 transcriptome

On the basis of the pre-experiment analysis, 1.0% RE was used as the test concentration (P10_RE), whereas the control did not contain the RE (P10_N). The strain P10 cultures incubated for 2 h were analyzed. Each culture was centrifuged at 3,500× g for 3 min at 4 °C and then they were immediately frozen in liquid nitrogen and stored at − 80 °C. Total RNA was extracted from the frozen strain P10 samples using the TRIzol reagent (Invitrogen, CA, USA). The RNA quality and quantity were determined using The Nano 6000 Assay Kit and the 2100 Bioanalyzer system (Agilent Technologies, Palo Alto, CA, USA). The RNA concentration was measured using the Qubit RNA Assay Kit and the Qubit 2.0 Fluorometer (Life Technologies, CA, USA). For each RNA sample, 3 µg was used along with the Ultra™ Directional RNA Library Prep Kit for Illumina (NEB, USA) to construct cDNA libraries, which were sequenced on the Illumina HiSeq™ 2500 platform (Novogene, Beijing, China). Paired-end reads were generated.

The raw data were stored in the fastq file format and clean data were obtained by removing reads containing adapters, reads containing poly-N sequences, and low-quality reads. Additionally, the Q20, Q30, and GC contents were calculated for the clean reads, which were then aligned to the *Burkholderia pyrrocinia* DSM10685 reference genome (GenBank accession number: GCA_001028665.1) using the Bowtie2 software (version 2.2.3). The HTSeq program (version 0.6.1) was used to analyze the paired-end clean reads to estimate gene expression levels. To quantify gene expression levels, the number of fragments per kilobase of exon per million fragments mapped (FPKM) was used. Differentially expressed genes were analyzed using DESeq2 (version 1.18.0) with |log_2_(Fold-Change)| > 1 and *P* < 0.05 set as the criteria for identifying significant DEGs. The enriched KEGG pathways among the DEGs were identified using KOBAS [64–66].

### Validation of DEGs by Q-RT-PCR

To confirm the transcriptome data were reliable, 12 DEGs identified by the RNA-seq analysis were selected for a Q-RT-PCR assay. Primers were designed using Primer-BLAST (Supplementary Table S4). Total RNA served as the template for the synthesis of first-strand cDNA using the StarScript II First-strand cDNA Synthesis Kit (GenStar BioSolutions (Beijing) Co., Ltd.). As an reference control, the housekeeping gene *recA* showed no variation in transcript abundance under the conditions tested. The expression of the 12 genes was analyzed using SYBR Green I (GenStar BioSolutions (Beijing) Co., Ltd.) and the CFX96 Touch Real-Time PCR System (BioRad, USA). The Q-RT-PCR started with a 2 min incubation at 95 °C, followed by 40 cycles consisting of 15 s at 95 °C and 30 s at annealing temperatures (Supplementary Table S4) and 30 s at 72 °C. Relative expression levels were calculated according to the 2^−ΔΔCt^ method. The Q-RT-PCR analysis was completed using three biological replicates.

### Analysis of the peanut RE components

The peanut RE components were analyzed using a gas chromatography system coupled with the Pegasus HT time-of-flight mass spectrometer (GC-TOF-MS) as previously described by Kind [67]. The system included a DB-5MS capillary column containing 5% diphenyl–95% dimethylpolysiloxane (30 m × 250 μm inner diameter, 0.25 μm film thickness; J&W Scientific, Folsom, CA, USA). The Chroma TOF 4.3X software (LECO Corporation) and the LECO-Fiehn Rtx5 database were used for extracting raw peaks, filtering and calibrating the baseline data, aligning peaks, performing the deconvolution analysis, identifying peaks, and integrating peak areas.

### Analysis of the effects of the peanut RE components on strain P10 biofilm formation

The LB medium was set as the control, whereas the LB medium supplemented with different concentrations (20 and 40 µmol/L) of amino acids (proline, glycine, and alanine) or organic acids (citric acid, oxalic acid, and malic acid) was used for the treatments.

The formation of the strain P10 biofilm was analyzed using a slightly modified version of the method described by Zhang et al. [60]. Briefly, strain P10 was grown in LB medium containing various peanut RE components at 30 °C with shaking (150 rpm) until the OD_600_ reached 1.0. The negative control comprised LB medium alone. Each treatment was replicated three times. The strain P10 suspensions (1:100 dilution) were added to the wells of polystyrene 96-well microtiter plates. After a 4-day static incubation at 30 °C, the non-adherent cells were removed and the wells were washed and dried naturally. Samples were stained in 1 mL 0.5% crystal violet for 20 min at room temperature. The excess crystal violet was removed and the wells were washed twice with distilled water. The bound crystal violet was solubilized using 1 mL ethanol:acetic acid solution (4:1 v:v). Biofilm formation was quantified on the basis of A_600_ measurements.

### Analysis of the effects of peanut RE components on the secretion of IAA by strain P10

Strain P10 was cultured in LB medium containing various peanut RE components at 30 °C with shaking (150 rpm) for 24 h. The culture was centrifuged at 12,000 × g for 10 min, after which 2 mL Salkowski reagent was added to 1 mL aliquots of the supernatant. Samples were incubated in darkness for 30 min at room temperature before determining the OD_530_. The IAA content of the supernatant was calculated as previously described [68].

### Statistical analysis

Differences among treatments were determined on the basis of an analysis of variance with Duncan’s multiple range test and Student’s *t-*test (*P* ˂ 0.05). The SPSS program (version 20.0) (IBM, Chicago, IL) was used for statistical analyses.

## Electronic supplementary material

Below is the link to the electronic supplementary material.

**Additional file 1**. Supplement Table. Table S1 Summary for the transcriptome assembly, Table S2 The FPKM of differential expressed genes (DEGs) in different samples, **Table S3** Composition of peanut root exudates analyzed by gas chromatography-mass spectrometry, **Table S4** Information of primers and gene used in this study, **Table S5** Reaction Procedure of Q-RT-PCR.

**Additional file 2**. Fig. S1 The expression fold change of 12 candidate genes of *Burkholderia pyrrocinia* P10 strain under root exduates of peanut.

## Data Availability

All data generated or analysed during this study are included in this published article.

## Abbreviations

PGPR: plant-growth promoting rhizobacteria
IAA: indole-3-acetic acid
REs: root exudates
LB: Luria-Bertani
OD: optical density
FPKM: fragments per kilobase of exon per million fragments
DEGs: Differentially expressed genes
KEGG: Kyoto Encyclopedia of Genes and Genomes
qRT-PCR: quantitative Real-Time PCR
GC-TOF-MS: gas chromatograph system coupled with a Pegasus HT time-of-flight mass spectrometer
CV: crystal violet
EPS: exopolysaccharide
GPMVs: giant plasma membrane vesicles
PIM-GMP: phosphomannose isomerase-guanosine 5’-diphospho-D-mannose pyrophosphorylase
QS: quorum sensing
MCP: methyl-accepting chemotaxis protein
T3SS: Type III protein secretion system
T6SS: Type VI secretion system
IAM: indole-3-acetamide pathway
TAM: tryptamine pathway
IpyA: Indole-3-pyruvate pathway
HIP-CoA: 3-[3aS, 4 S, 7aS)-7a-methyl-1,5-dioxo-octahydro- 1 H-inden-4-yl]propanoyl-CoA
ABC: ATP-binding cassette
ABCC: ATP binding cassette subfamily C
TCA: tricarboxylic acid
