# Supplementary material for: The peanut root exudate increases the transport and metabolism of nutrients and enhances the plant growth-promoting effects of burkholderia pyrrocinia strain P10
Source: BMC Microbiol. 2023 Mar 30;23:85. doi: 10.1186/s12866-023-02818-9 (PMC10061817; doi:10.1186/s12866-023-02818-9)
Supplement: Supplementary file 2 — Additional file 2. Fig. S1 The expression fold change of 12 candidate genes of Burkholderia pyrrocinia P10 strain under root exduates of peanut. [file 12866_2023_2818_MOESM2_ESM.doc]

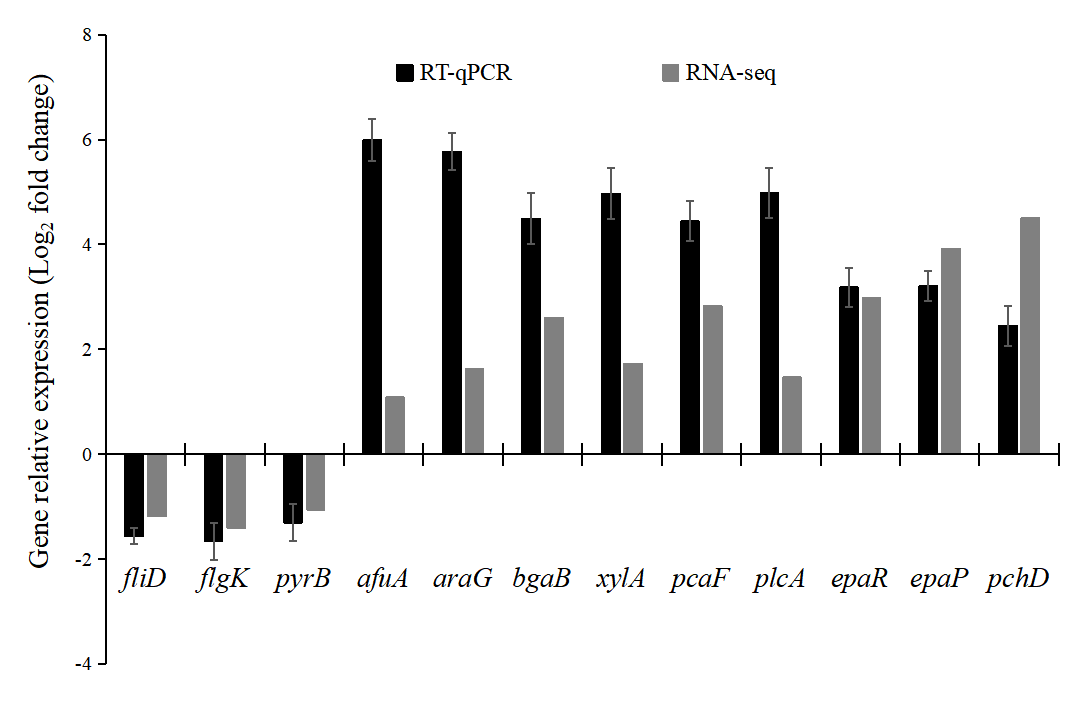


Supp Fig. 1 The expression fold change of 12 candidate genes of *Burkholderia pyrrocinia*

P10 strain under root exduates of peanut.

Transcription levels of P10 12 genes were obtained by qRT-PCR from P10_RE. Expression levels are represented as Log2(Fold change) relative values in camparison to the expression levell of P10_N. All the results are from three independent experiments. Canidate genes mainly included Filament cap gene *fliD*, flagellar hook-associated protein gene *flgK*, aspartate carbamoyltransferase catalytic subunit gene *pyrB*, iron(III) transport system substrate-binding protein gene *afuA*, L-arabinose transport system ATP-binding protein gene *araG*, beta-galactosidase gene *bgaB*, xylose isomerase gene *xylA*, acetyl-CoA acyltransferase gene *pcaF*, phospholipase C gene *plcA*, type III secretion protein T gene *epaR*, type III secretion protein R gene *epaP*, salicylate---[aryl-carrier protein] ligase gene *pchD*.
